# UCMAC: A Cooperative MAC Protocol for Underwater Wireless Sensor Networks

**DOI:** 10.3390/s18061969

**Published:** 2018-06-19

**Authors:** Hee-won Kim, Tae Ho Im, Ho-Shin Cho

**Affiliations:** 1 School of Electronics Engineering, Kyungpook National University, Daegu 41566, Korea; hwkim@ee.knu.ac.kr; 2 Oceanic IT Convergence Technology Research Center, Hoseo University, Asan 31499, Korea; taehoim@hoseo.edu

**Keywords:** underwater wireless sensor network, automatic repeat request, medium access control, cooperative communication, spatial diversity, cooperative region, cooperative ARQ, cooperative MAC

## Abstract

This paper proposes a cooperative medium access control (MAC) protocol for underwater wireless sensor networks (UWSNs) named UCMAC, which fundamentally benefits from cooperative communication. In UCMAC, a source identifies cooperators and provides its destination with a list of the cooperators while also delineating their proximity to the destination. For erroneous reception of data packets, the destination then requests retransmission to the cooperators in a closest-one-first manner. A designated cooperator transmits the buffered data packet it has successfully overheard from the source or other cooperators. A signaling procedure and the various waiting times of the nodes are carefully designed to address the overheads that stem from cooperation. Through computer simulation, this paper evaluates UCMAC in terms of system throughput, latency, single-hop packet delivery ratio (PDR), and energy efficiency. The results show that UCMAC performs better than existing schemes, including MACA-U and CD-MACA.

## 1. Introduction

In recent years, researchers have actively studied numerous applications of underwater wireless sensor networks (UWSNs), including tactical surveillance, disaster prevention, and oceanographic observation [1]. In particular, studies have proposed an array of medium access control (MAC) protocols to enable communicating nodes to access the shared underwater channel. Compared to terrestrial radio signals, underwater acoustic signals suffer from high attenuation, long propagation delays, severe multipath fading, and high bit error rates (BER) in the channel. Achieving reliable delivery of data packets under such poor channel conditions necessitates the use of the automatic repeat request (ARQ) and/or the forward error correction (FEC) techniques at the link layer.

**Figure 1:**
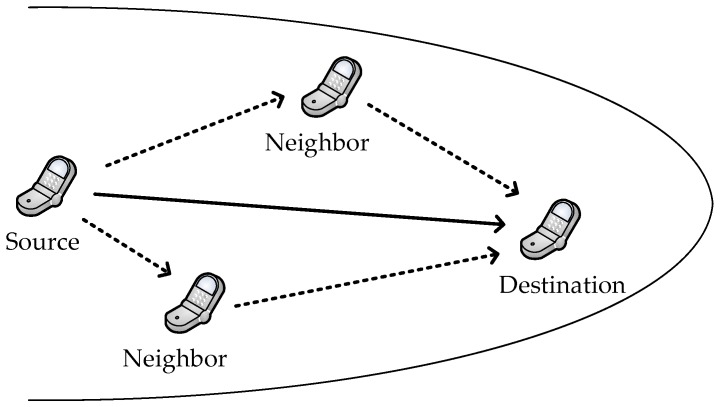
Cooperative communication.

Traditionally, ARQ methods are divided into three categories: stop-and-wait (S&W), go-back-n (GBN), and selective repeat (SR) [2]. In the S&W method, a transmitter waits for an acknowledgement (ACK) from a receiver after transmitting a data packet and then retransmits the data packet if it fails to receive the ACK. Many existing MAC protocols for the UWSNs [3,4,5] employ the S&W method due to its simplicity and suitability for half-duplex channels. However, long propagation delays in the acoustic signals make the S&W method inefficient because the sender’s lengthy waiting time wastes resources. In the GBN and SR methods, on the other hand, senders can transmit data packets continuously while waiting for receivers’ ACKs. This can increase network throughput at the expense of receiver processing complexity, but it requires the full-duplex system for concurrent delivery of ACK packets, which has yet to prove workable in the bandwidth-limited underwater channel. Another new approach is the so-called *cooperative ARQ*, which is based on the cooperative communication technique. The basic idea of the cooperative communication technique is that neighbor nodes (cooperators) can provide an alternative path for other pairs of communicating nodes (source–destination), as shown in Figure 1. This generates spatial diversity by enabling the transmission of independent copies of the signal, thus allowing independently faded versions of the signal at the destination [6].

Earlier research on cooperative communication—mainly conducted in the terrestrial domain—focused on issues such as theoretical analysis based on information theory and its implementation aspects [7,8]. Such analyses led to the creation of link layer protocols to gain benefits by coordinating nodes thereafter. Some studies [9,10] proposed a cooperative ARQ scheme that simply describes the behavior of the nodes for cooperative retransmission, analyzing performance using their own analysis model, while others [11,12,13,14,15,16,17,18,19,20,21] proposed a so-called *cooperative MAC protocol* that mostly combines the cooperative ARQ mechanism with the MAC protocol. Depending on the cooperative mechanism, the cooperative MAC protocols can be categorized as a *proactive* type, a *reactive* type, or a *hybrid* type. In proactive-type protocols, the cooperation process mostly starts before a destination receives an initial data packet from a source, whereas reactive-type protocols are an on-demand method where the destination requests cooperation only when it fails to receive the data packet. A major problem with proactive-type protocols is that cooperators may send a redundant data packet to the destination even when the destination succeeds in receiving the initial packet from the source. This redundancy leads to a waste of energy but can decrease delay with a high chance of successful destination decoding. On the other hand, the reactive-type protocols can save energy at the expense of delay. Both features of the proactive- and reactive-type protocols combine to form hybrid-type protocols.

Existing cooperative MAC protocols for terrestrial wireless networks are largely based on the distributed coordination function (DCF) of the IEEE 802.11 standard [22], known as carrier sense multiple access with collision avoidance (CSMA/CA). In addition, for cooperator selection, these protocols largely rely on channel state information (CSI) between nodes. However, due to intrinsic channel characteristics and ever-changing channel states, these protocols do not work well in the underwater environment. In other words, utilizing the carrier sensing technique and keeping the CSI between all neighboring nodes up to date in the underwater channel are difficult. Until now, relevant research has taken into account such constraints to deal with several important issues such as cooperative signaling strategies [23], criteria for best cooperator selection [24,25,26] and cooperative routing protocols [27,28]. To the best of our knowledge, however, researchers have not yet actively examined the cooperative MAC protocol in the underwater channel. Unlike the terrestrial case, underwater networking suffers from so-called *space–time uncertainty* problem due to intrinsic channel characteristic of the long propagation delay [29]. The space–time uncertainty opens up new aspect on packet collision in the underwater channel, whereas this is irrelevant to terrestrial networks as the propagation delay is negligibly small and can be ignored. Therefore, we are motivated to devise a novel underwater-specific cooperative MAC protocol that can perform efficiently even with the space–time uncertainty.

Based on our previous work [30,31], we propose a reactive-type cooperative MAC protocol for the UWSNs, named Underwater Cooperative MAC (UCMAC). A source identifies cooperators and provides the destination with a list of cooperators along with information about their proximity to the destination. For erroneous reception of data packets, the destination then requests retransmission to the cooperators in a closest-one-first manner. A designated cooperator transmits the buffered data packet stored just in case to the destination. If no available cooperators exist, UCMAC follows the conventional MACA-U protocol [32] that basically uses 3-way handshaking (RTS–CTS–DATA) and optionally adds ACK without cooperation process. We evaluate the proposed scheme in terms of system throughput, latency, single-hop packet delivery ratio (PDR), and energy efficiency, comparing it to MACA-U and CD-MACA [12].

The novelty of this paper lies in the design of signaling procedure and in the calculation of the appropriate timer lengths of nodes to benefit from the cooperative diversity gains while overcoming the space–time uncertainty problem in the underwater channel. Also, we escape potential collisions between cooperators by making them cooperate one at a time and define a cooperative region to involve only qualified cooperators in cooperation process.

The remainder of this paper is organized as follows: in Section 2, we review some related work. After describing a system model in Section 3, we explain how the UCMAC works in Section 4. Section 5 analyzes UCMAC’s performance through computer simulation. Finally, we conclude the paper by presenting additional work in Section 6.

## 2. Related Work

Previous studies have proposed many cooperative MAC protocols for terrestrial wireless networks. In [14], Liu et al. presented a proactive type of the cooperative MAC protocol called CoopMAC. Based on the capability of rate adaptation at the physical (PHY) layer, a low-rate source first decides whether data forwarding through a high-rate cooperator (source–cooperator–destination) reduces delay compared to direct transmission (source–destination). Then, the source invokes the cooperative mode instead of the IEEE 802.11 protocol only if doing so could decrease the delay. The point is that each node should overhear all transmissions and continuously update a neighbor table in which the rate information of all neighbors is stored. Because this would be demanding in the changeable underwater channel, UCMAC simply uses propagation delay information instead of keeping the table. In addition, CoopMAC uses the S&W ARQ, which means that the destination does not request any cooperation from the cooperators when it fails to receive a data packet. A reactive-type protocol, cooperative diversity–multiple access with collision avoidance (CD-MACA), was proposed in [12]. In CD-MACA, cooperators buffer overheard data and then transmit it to a destination when overhearing a clear-to-send (CTS) packet that corresponds to the data. This gives the destination more chances to successfully decode its received data packet with independent samples. However, in the underwater channel where carrier sensing is less effective, such diversity gain may not be obtained because of packet collision among the data packets transmitted by the source and the cooperators. Another issue is that even the nodes that have inferior channel qualities to the destination cooperate. For efficient cooperation, cooperator selection criteria should be considered. Note that, in CoopMAC, the cooperator through which a minimum delay occurs is selected as the sole cooperator. To alleviate the need to identify the best cooperator each time the source transmits data, persistent relay CSMA (PRCSMA) [15] enables a set of the most appropriate nodes near to the destination to become cooperators. If the destination fails in decoding a data packet, the cooperators persistently retransmit data packets until the destination succeeds or the cooperation phase ends. However, loss of ACK packets forces the cooperators to continue sending the data packets even after the destination’s successful decoding. Moreover, they know whether to cooperate or not only after receiving a claim-for-cooperate (CFC) packet transmitted by the destination, which makes all neighbors of the source store overheard data in their memory banks. UCMAC eliminates these redundancies by requesting the retransmission from the cooperators one at a time and defining a cooperative region where nodes cooperate. The authors have also proposed DQCOOP [17], which is based on their previous work, DQCA [33] and its different version, DQMAN [34]. In DQCOOP, the contention window is divided into slots so that each cooperator randomly selects one slot for the channel access. Then the destination feeds information of access success or failure back to the cooperators, which retransmit their data packets accordingly. This can prevent data packet collision, but every node must be tightly synchronized and maintain special queues and variables. UCMAC requires neither time synchronization nor special queues and variables. In [19], Antonopulous et al. proposed a network coding-based cooperative MAC protocol named NCCARQ-MAC that applies network coding technique at the MAC layer perspective. Similar to CD-MACA, every node keeps a copy of all data packets overheard in preparation for cooperation. In NCCARQ-MAC, a destination piggybacks its data (if it exists) on a request-for-cooperation (RFC) packet transmitted to request retransmission of the source’s data. Then a cooperator creates a network coded-packet with the source’s and destination’s data packets and then transmits it to the source and the destination, which allows them to obtain the respective data quickly. Taking into account the impact of PHY layer, the performance of NCCARQ-MAC is further investigated and analyzed under realistic channel conditions, especially for correlated shadowing [20,21]. In that context, network coding technique contributes to increase in network throughput when bidirectional traffic is dominantly generated. Such technique may be inefficient in sensor networks where unidirectional traffic dominates. Reference [16] proposed a *hybrid*-type cooperative MAC protocol where both of the proactive and the reactive mechanisms are applied. Graded back-off time that depends on a maximum achievable rate makes only the highest-rate cooperator cooperate alone (proactive). If data packet decoding is unsuccessful, the destination sends a negative ACK (NACK) packet to its source and/or the cooperator to request data retransmission (reactive). Prior to the retransmission, handshake of request-to-send (RTS) and CTS packets is performed again. In the UCMAC, a cooperator directly retransmits a data packet eliminating this handshaking procedure to reduce delay.

Meanwhile, existing related work for the underwater wireless networks has dealt with several important issues other than MAC layer ones. In [23], Han et al. proposed a new signaling method called wave cooperative (WC) transmission where a cooperator immediately amplifies and forwards source signals whenever their multipath components pass through. The WC method can outperform existing methods such as the amplify-and-forward (AF) and the decode-and-forward (DF) because a destination can receive high strength multipath components in less time. Reference [24] developed a best-relay (or cooperator) selection criterion, called cooperative best relay assessment (COBRA) to select best cooperators under the varying underwater channel. Rather than using the CSI, the COBRA relies on propagation delays between any pair of nodes in the network and statistical channel parameters when selecting the best cooperator. The researchers in [24] briefly mentioned a methodology for MAC based on RTS–CTS handshaking and the COBRA criterion. Clearly, it is necessary for the cooperative MAC protocols to utilize the RTS–CTS handshaking procedure in acquiring and/or sharing cooperator information.

## 3. System Description

We assume that all nodes, which are equivalent in terms of capability and physical specification, are fixed on the seabed without timing synchronization and that they acquire propagation delay information from/to one-hop-distance neighbors through network initialization [35]. In addition, the nodes configure a distributed and partially connected mesh topology where they are connected to their one-hop distance neighbors. Each node can communicate directly with any of its one-hop distance neighbor. Regarding the means of cooperation, the DF signaling strategy [6] is applied, and a cooperator selectively joins the retransmission of erroneous packets as a replacement of source. We also assume that bit errors happen only in the data payload, not in control messages such as header and control packets, which are much smaller than the payload. In regard to the channel model, the empirical underwater acoustic channel model [36] is considered, which is in wide use in the literature. In this model, the underwater channel is characterized by attenuation that increases with signal frequency, noise of which power spectral density decays with the frequency, and signal-to-noise ratio (SNR) that varies over the signal bandwidth, etc.

## 4. Operation of Proposed Protocol

**Figure 2:**
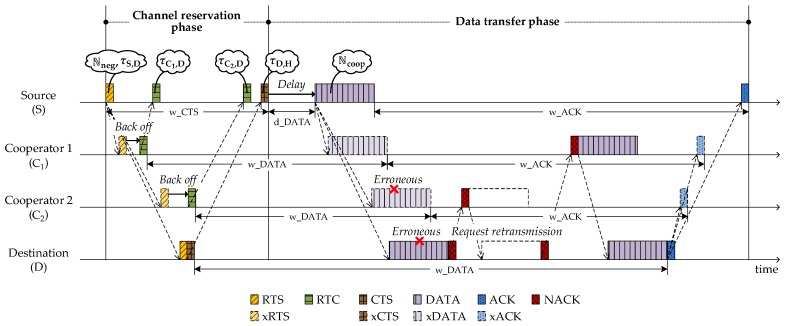
Basic procedure of UCMAC.

Figure 2 illustrates a basic UCMAC procedure with two cooperators ($C_{1}$ and $C_{2}$) that are located between the source (S) and destination (D). UCMAC consists of two phases, *channel reservation* and *data transfer*. In the channel reservation phase, the source reserves the channel and identifies the cooperators through their control packet responses. In the data transfer phase, the source transmits a data packet (DATA) attaching a list of cooperators sequenced by closeness to the destination. Then, referring to the list, the destination requests retransmission of erroneous packets to cooperators in the closest-one-first manner. Meanwhile, the cooperators store the data packet (denoted by xDATA in Figure 2) that is overheard during the source’s transmission. The parameters carried by packets are denoted inside speech bubbles as shown in Figure 2 and the details along with time parameters like w_ACK and w_DATA are explained in the corresponding subsections. Table 1 defines some of the parameters in advance to make it easier to understand subsequent sections.

### 4.1. Channel Reservation Phase

The source begins with the channel reservation by sending a *request-to-send* (RTS) to the destination, which contains the propagation delay between source and destination, $\tau_{S,D}$. Overhearing the RTS, a neighbor *n* determines whether it is an eligible cooperator based on the following criteria:

(1) $$\tau_{S,n}<\tau_{S,D}\;\mathrm{and}\;\tau_{n,D}<\tau_{S,D}$$

**Figure 3:**
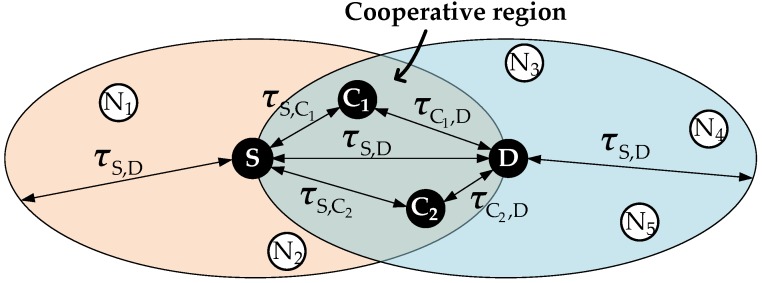
Cooperative region.

This means that the cooperator should be closer to both the source and the destination than the source-to-destination distance. Accordingly, the *cooperative region* is defined as the area where eligible cooperators may exist, as shown in Figure 3. Every neighbor *n* that resides in the cooperative region sends a *request-to-cooperate* (RTC) containing $\tau_{n,D}$, while the destination sends a *clear-to-send* (CTS) back to the source in response to the RTS. Then, referring to the RTC, the source builds a list of cooperators, ${\mathbb{N}}_{\mathrm{coop}}$, which is sequenced based on their closeness to the destination. To prevent excessively long operation time resulting from the participation of the cooperators, the number of cooperators included in ${\mathbb{N}}_{\mathrm{coop}}$ is limited by $N_{\mathrm{coop}}^{\max}$. Supposing $\tau_{C_{2},D}$ is smaller than $\tau_{C_{1},D}$ in Figure 3, the destination gives selection preference to $C_{2}$. If the retransmssion of $C_{2}$ also fails, the retransmission continues through the next most preferred cooperator, $C_{1}$, in this example. If no available cooperator exists, then the conventional MACA-U protocol works.

**Figure 4:**
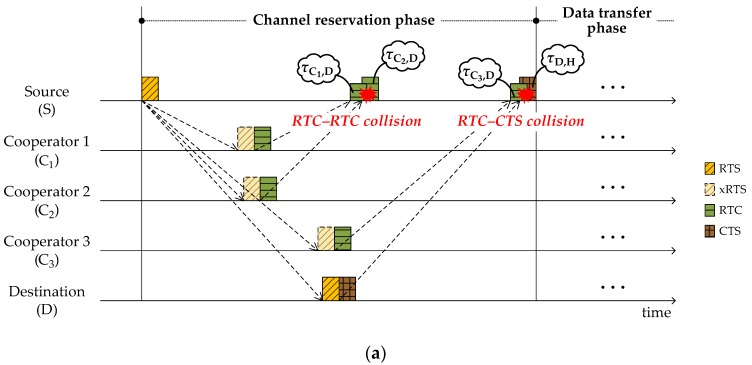

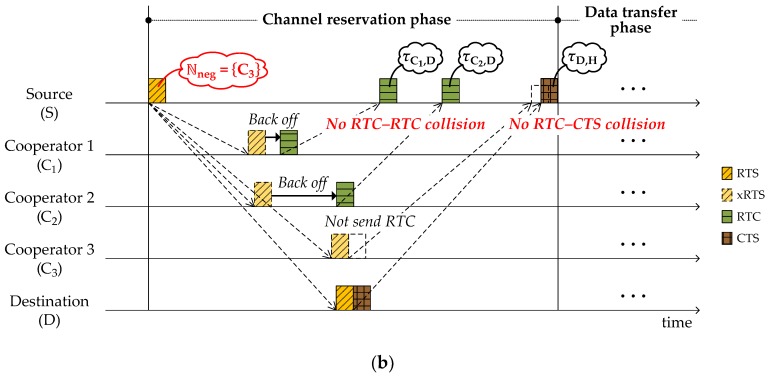
RTC–RTC and RTC–CTS collision cases: (**a**) without any countermeasures; (**b**) with backoff and prohibition of RTC transmission.

RTC and CTS, the responses to the RTS from cooperators and the destination, respectively, may collide at the source. Figure 4a shows examples of RTC–RTC and RTC–CTS collisions. RTC–CTS collisions are significantly more severe than RTC–RTC collisions; they trigger the whole procedure to restart from the beginning. To avoid RTC–CTS collisions, we prohibit the neighbors that may cause collisions from transmitting RTC. Based on the pre-acquired knowledge regarding propagation delay information, the source builds a list of potential RTC–CTS collision-causing neighbors, ${\mathbb{N}}_{\mathrm{neg}}$, and includes it into RTS. Then the neighbors included in ${\mathbb{N}}_{\mathrm{neg}}$ are banned from sending RTC. In Figure 4b, $C_{3}$ represents a case of RTC transmission prohibition so that CTS can escape a collision at the source. Meanwhile, RTC–RTC collisions can be alleviated by implementing random backoff times.

### 4.2. Data Transfer Phase

**Figure 5:**
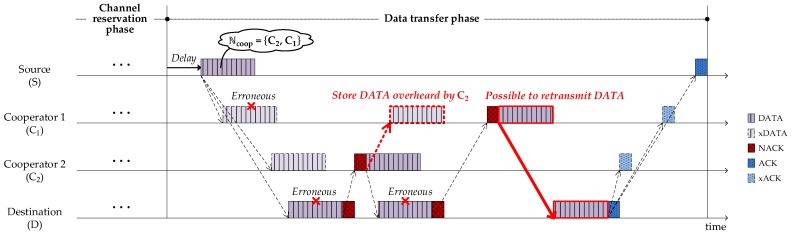
Enabling cooperation through additional DATA overhearing.

In Figure 5, the source sends the deferred DATA to the destination, which contains ${\mathbb{N}}_{\mathrm{coop}}$. The next section explains the reason for deferment. Overhearing the DATA, the neighbors included in ${\mathbb{N}}_{\mathrm{coop}}$ recognize themselves as cooperators and hold the successfully decoded data payload in case a request for retransmission is sent. Those not included in ${\mathbb{N}}_{\mathrm{coop}}$ are exempted from the cooperation. The possible reasons for not being included in ${\mathbb{N}}_{\mathrm{coop}}$ are:

- The source fails to receive RTCs.
- The size of ${\mathbb{N}}_{\mathrm{coop}}$ reaches the limit, $N_{\mathrm{coop}}^{\max}$.

If the payload is successfully decoded, the destination sends ACK back to the source. Otherwise, the destination requests retransmission by continuously sending NACK to the closest available cooperator specified in ${\mathbb{N}}_{\mathrm{coop}}$ until the retransmission is successful or ${\mathbb{N}}_{\mathrm{coop}}$ is exhausted. Once the retransmission is successful, the destination sends ACK to the source. Overhearing the ACK, the cooperators recognize the completion and discard the stored data.

As Figure 5 shows, even when cooperators ($C_{1}$ in this example) fail at first to overhear DATA from the source, they can recover the failed DATA by overhearing other cooperators’ ($C_{2}$ in this example) retransmission and therefore can join the retransmission (denoted by bold arrow) with the recovered DATA.

### 4.3. Waiting Times

The source, the cooperators, and the destination each manages a waiting-mechanism to escape undesirable collisions. In this section, we calculate the waiting times that must be very carefully set to make the proposed scheme work properly.

#### 4.3.1. Waiting Times at Source

**Figure 6:**
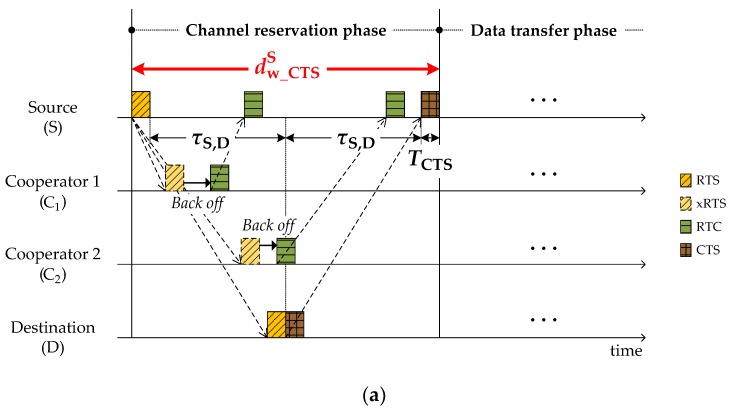

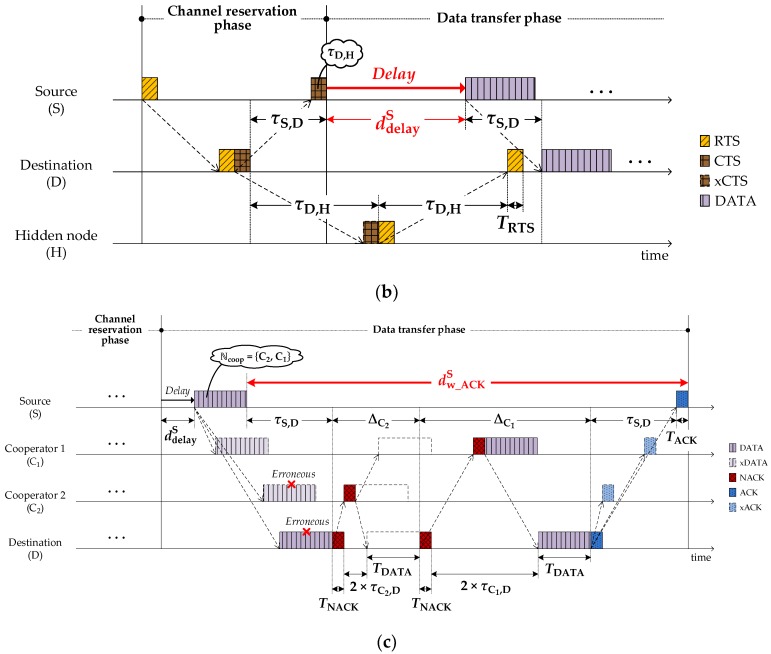
Decision of waiting time at source: (**a**) $d_{w\_\mathrm{CTS}}^{S}$; (**b**) $d_{\mathrm{delay}}^{S}$; (**c**) $d_{w\_\mathrm{ACK}}^{S}$.

The source manages two kinds of waiting time and one deferment as shown in Figure 6. In the *channel reservation* phase, after transmitting RTS, the source waits for CTS from the destination during the period of:

(2) $$d_{w\_\mathrm{CTS}}^{S}=2\times \tau_{S,D}+T_{\mathrm{CTS}}$$

which is easily obtained from Figure 6a. After receiving CTS, the source intentionally delays DATA transmission to avoid a collision with RTS sent by a hidden node (H) who starts a new communication with the destination as shown in Figure 6b. The amount of delay required is denoted by $d_{\mathrm{delay}}^{S}$. To avoid the RTS–DATA collision, the DATA should be scheduled to arrive after the possible RTS arrival (Figure 6b). That is:

(3) $$2\times \tau_{S,D}+d_{\mathrm{delay}}^{S}>2\times \tau_{D,H}+T_{\mathrm{RTS}}$$

where the $\tau_{D,H}$ specified in CTS is a propagation delay between the destination and its farthest neighbor who may be hidden from the source. By rearranging Equation (3), $d_{\mathrm{delay}}^{S}$ is obtained by:

(4) $$d_{\mathrm{delay}}^{S}=\min\left(2\times (\tau_{D,H}-\tau_{S,D})+T_{\mathrm{RTS}},\;0\right).$$

After DATA transmission, the source waits for ACK from the destination. The waiting time for ACK might be augmented according to the number of cooperators participating in the retransmission. The time segment augmented by cooperators *j* is given by (Figure 6c):

(5) $$\Delta_{C_{j}}=2\times \tau_{C_{j},D}+T_{\mathrm{NACK}}+T_{\mathrm{DATA}}.$$

Thus, the maximum time spent by the source until receiving ACK is:

(6) $$d_{w\_\mathrm{ACK}}^{S}=\left(2\times \tau_{S,D}+T_{\mathrm{ACK}}\right)+\underset{C_{j}\in {\mathbb{N}}_{\mathrm{coop}}}{\sum }\Delta_{C_{j}}.$$

If CTS and ACK are not received within the aforementioned time periods $d_{w\_\mathrm{CTS}}^{S}$ and $d_{w\_\mathrm{ACK}}^{S}$, respectively, the source restarts the whole procedure beginning with sending a new RTS after a random backoff time that follows the binary exponential backoff (BEB) algorithm.

#### 4.3.2. Waiting Times at Cooperators

**Figure 7:**
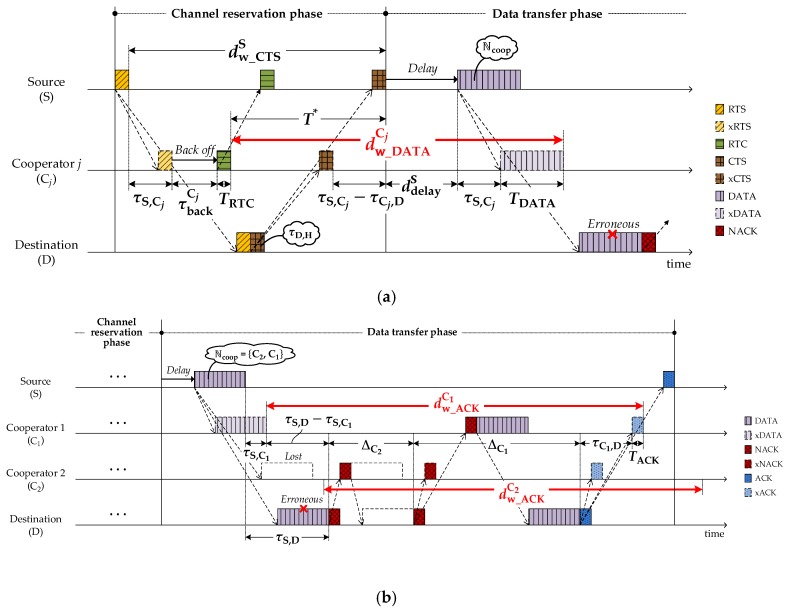
Decision of time-parameters at cooperator *j*: (**a**) $d_{w\_\mathrm{DATA}}^{C_{j}}$; (**b**) $d_{w\_\mathrm{ACK}}^{C_{j}}$.

As shown in Figure 7a, the cooperator *j* takes a random backoff ($\tau_{\mathrm{back}}^{C_{j}}$) before transmitting RTC, which is intended to prevent RTC–RTC collisions. The cooperator *j* then stays silent to avoid causing any collision with the source–destination communication. In order to estimate the silence duration, the cooperator *j* needs to know how many cooperators could be engaged in the communication by reading the ${\mathbb{N}}_{\mathrm{coop}}$ carried in DATA, so, the cooperator *j* waits to overhear the DATA during the period of:

(7) $$d_{w\_\mathrm{DATA}}^{C_{j}}=T^{*}={\hat{d}}_{\mathrm{delay}}^{S}+\tau_{{S,C}_{j}}+T_{\mathrm{DATA}}$$

where:

(8) $$T^{*}=d_{w\_\mathrm{CTS}}^{S}-\left(\tau_{{S,C}_{j}}+\tau_{\mathrm{back}}^{C_{j}}+T_{\mathrm{RTC}}\right),$$

and ${\hat{d}}_{\mathrm{delay}}^{S}$ is a modified value of $d_{\mathrm{delay}}^{S}$ which is given in Equation (4). Due to the lack of knowledge of $\tau_{D,H}$ at the moment, the cooperator guesses $d_{\mathrm{delay}}^{S}$ as the maximum value of $\tau_{\max}$. Then, according to the number of cooperators engaged, the cooperator extends the silence duration in which the cooperator itself possibly participates in retransmission. Figure 7b shows the extended silence duration of the cooperator *j* after the expiration of $d_{w\_\mathrm{DATA}}^{C_{j}}$, which is given by:

(9) $$d_{w\_\mathrm{ACK}}^{C_{j}}=\left(\tau_{S,D}-\tau_{{S,C}_{j}}+\tau_{C_{j},D}+T_{\mathrm{ACK}}\right)+\underset{C_{j}\in {\mathbb{N}}_{\mathrm{coop}}}{\sum }\Delta_{C_{j}}.$$

In case the cooperator fails to overhear DATA, the maximum number of cooperators ($N_{\mathrm{coop}}^{\max}$) is used to calculate the second term of Equation (9).

#### 4.3.3. Waiting Times at Destination

**Figure 8:**
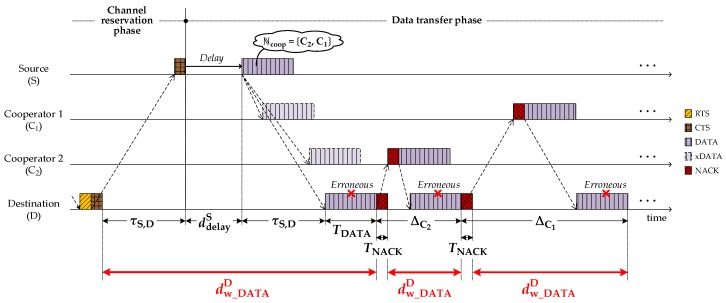
Decision of $d_{w\_\mathrm{DATA}}^{D}$ at destination.

After sending CTS or NACK, the destination waits for DATA arrival. As shown in Figure 8, the waiting time varies according to the packet type (CTS or NACK) by:

(10) $$d_{w\_\mathrm{DATA}}^{D}=\{\begin{array}{ll} 2\times \tau_{S,D}+d_{\mathrm{delay}}^{S}+T_{\mathrm{DATA}}, & \mathrm{after}\;\mathrm{sending}\;\mathrm{CTS}\;\mathrm{to}\;\mathrm{source}\\ \;\Delta_{C_{j}}-T_{\mathrm{NACK}}, & \mathrm{after}\;\mathrm{sending}\;\mathrm{NACK}\;\mathrm{to}\;\mathrm{cooperator}\;j. \end{array}$$

If no cooperators exist, the destination does not request any cooperation and returns to an idle state.

## 5. Performance Evaluation

Through the computer simulation, we compare UCMAC with the conventional MACA-U [32] and CD-MACA [12] in terms of system throughput, latency, energy consumption, and single-hop PDR. MACA-U is the non-cooperative underwater-specific MACA [37] protocol that basically uses 3-way handshaking (RTS–CTS–DATA) and optionally adds ACK at the end. In our evaluation, we consider the MACA-U with ACK to make the comparison fair. CD-MACA has a simple cooperation mechanism that allows neighboring nodes to opportunistically participate in retransmission.

### 5.1. Simulation Model

In a grid network, 36 static nodes are located around each grid point with 10% variation in the spacing. Each node generates data traffic that follows the Poisson arrival process with the rate of λ and operates in half-duplexing mode, which may cause the busy terminal problem [38]. To reflect the characteristics of underwater channel, we apply the empirical underwater acoustic channel model [36]. Since we use no error correction technique, DATA with even a single bit error is assumed to be erroneous, while other control packets are assumed to be error-free. We determine the data rate and the transmission/receiving (Tx/Rx) powers by referring to the specifications of the commercial Teledyne Benthos ATM-903 underwater modem [39]. For simplicity, we ignore power consumption in idle mode. For CD-MACA, we set the duration time the cooperator holds DATA and the memory size to 30 s and 100 packets, respectively. Table 2 lists the default values of the system parameters used in the simulation.

### 5.2. Simulation Results

First of all, we define performance metrics as follows:

- System throughput: The average number of DATA bits successfully received by the intended destinations per second (measured in bps)
- Latency: The average time interval between generation and successful delivery of DATA packets at the intended destinations (measured in s)
- Single-hop PDR: The ratio of the number of DATA packets successfully delivered at the intended destinations to the total number of DATA packets generated (measured in %)
- Energy efficiency: The average number of DATA bits successfully received by the intended destinations per Joule (measured in bits/J)

**Figure 9:**
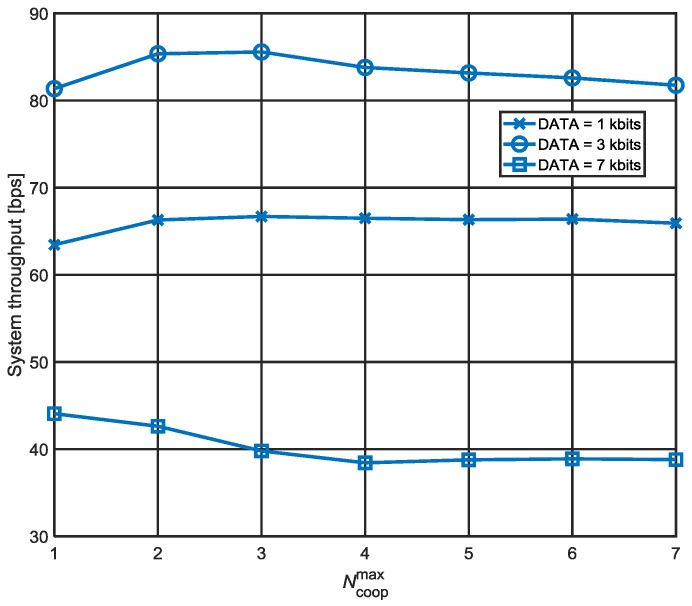
System throughput versus $N_{\mathrm{coop}}^{\max}$ (λ=0.002).

Figure 9 demonstrates the existence of an appropriate $N_{\mathrm{coop}}^{\max}$ in terms of the system throughput for various DATA sizes. It is generally accepted to assume that high cooperative diversity gains lead to a high system throughput. If $N_{\mathrm{coop}}^{\max}$ is small, the cooperative diversity gains may not be maximally obtained even though only a small amount of overheads is produced by additional cooperation process at the same time. On the other hand, a high $N_{\mathrm{coop}}^{\max}$ makes excessive overheads exceed the cooperative diversity gains. Consequently, an appropriate level of $N_{\mathrm{coop}}^{\max}$ exists depending on network circumstances. For example, when the DATA size is 3 kbits, we expect that $N_{\mathrm{coop}}^{\max}$ of 3 results in the highest system throughput.

Meanwhile, the optimal $N_{\mathrm{coop}}^{\max}$ also varies with the data size. As the data packet size becomes larger, the susceptibility to packet error increases. Therefore, if the data size is too large, the data packets retransmitted by cooperators are also likely to be erroneous. In this situation, increasing $N_{\mathrm{coop}}^{\max}$ just brings about increase in overheads. On the contrary, a small data packet is more likely to be successfully received, and therefore, a relatively higher $N_{\mathrm{coop}}^{\max}$ is allowed at the expense of additional overheads.

**Figure 10:**
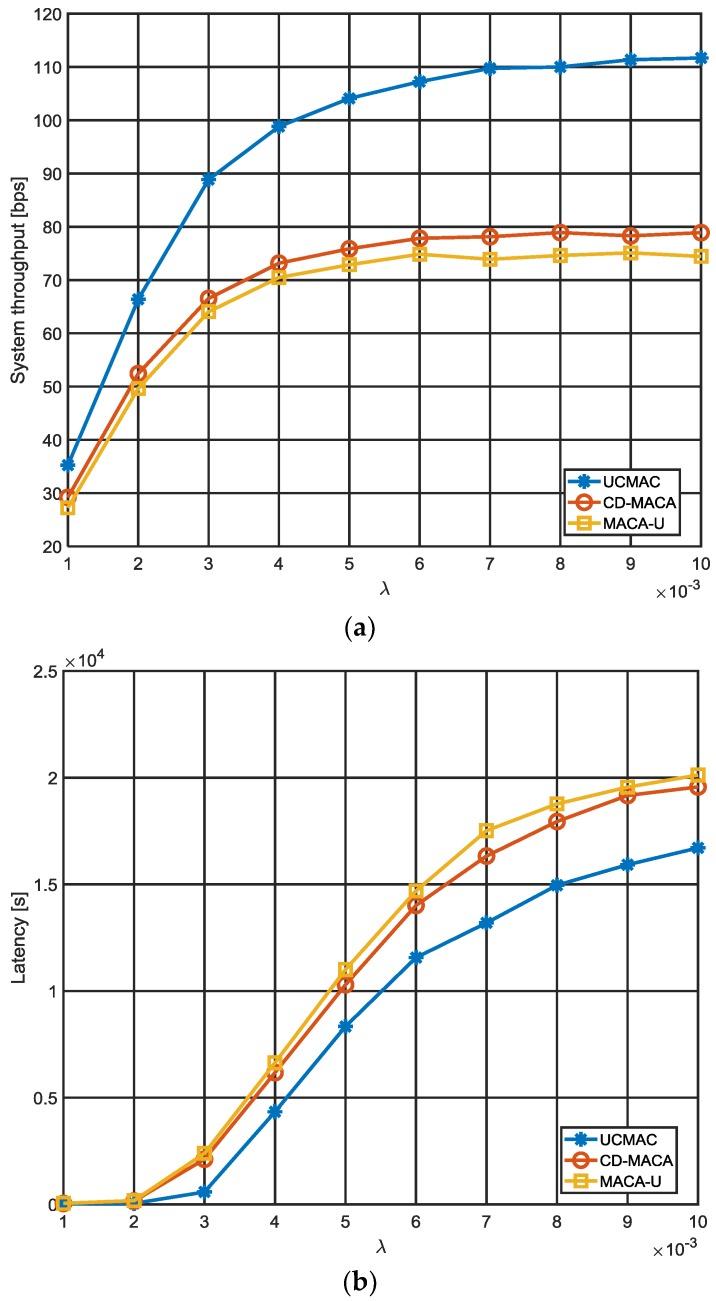

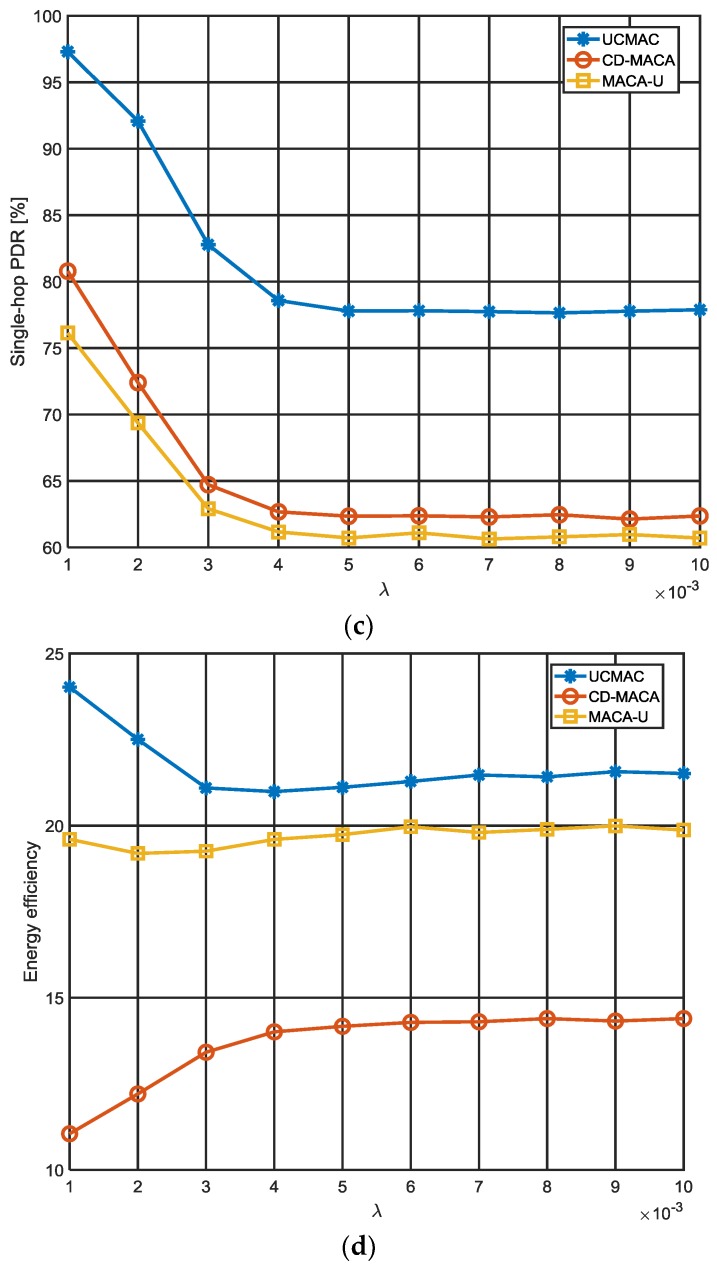
Performance comparison with comparing schemes ($N_{\mathrm{coop}}^{\max}=2$, DATA size = 1 kbits): (**a**) System throughput; (**b**) Latency; (**c**) Single-hop PDR; (**d**) Energy efficiency.

In Figure 10, we compare UCMAC with MACA-U and CD-MACA by setting $N_{\mathrm{coop}}^{\max}$ and DATA size at 2 and 1 kbits, respectively. Overall, UCMAC outperforms the other schemes as a result of its well-coordinated cooperation process. MACA-U, which is non-cooperative, shows relatively low performances compared to cooperative protocols. First, our analysis shows that UCMAC offers much better system throughput than other schemes (Figure 10a). This is because retransmitted DATAs arrive at the destinations more quickly with the aid of the well-coordinated cooperators. Although CD-MACA also benefits from cooperation, such cooperative gains are smaller than those of UCMAC mainly due to the lack of coordination between the cooperators. CD-MACA performs slightly better than MACA-U, allowing neighboring nodes or cooperators to retransmit DATA opportunistically. In MACA-U’s case, when packet errors happen, the destinations do not request any cooperation and just rely on source retransmissions. If packet errors persist, each source is more likely to take a longer backoff time, which will result in larger decreases in the system throughput. The result of latency can be understood in the same context (Figure 10b). In CD-MACA, packet collisions may occur between DATAs retransmitted by the cooperators; UCMAC escapes such collisions by making the cooperators retransmit DATA one at a time. This increases the likelihood that the intended destinations successfully receive DATAs after a smaller number of retransmission, thus reducing the latency. At the same time, elimination of packet collisions also helps UCMAC to achieve the high single-hop PDR (Figure 10c). This means that cooperation is highly effective in the delivery of DATAs even when the traffic is heavy, and UCMAC can be reliable under any circumstances. In CD-MACA, more DATAs end up being discarded due to collisions, which results in the lower single-hop PDR in spite of cooperation. Obviously, non-cooperative MACA-U has the lowest single-hop PDR as the destinations rely solely on their own sources. In terms of energy consumption, our analysis also shows that UCMAC is most efficient (Figure 10d).

Unlike CD-MACA where all neighbors commonly included in both source and destination coverages serve as cooperators without considering whether they have better channel-to-destination than sources, UCMAC selects only beneficial neighbors in terms of channel quality as cooperators and accordingly saves energy by using lower transmission power and fewer retransmissions.

Furthermore, since no packet collisions occur between retransmitted DATAs as aforementioned, energy consumption can be significantly reduced. The reason CD-MACA is more energy-intensive than MACA-U is because all cooperators wastefully participate in every retransmission in CD-MACA. The decrease in energy efficiency of UCMAC in the range of low λ largely stems from the extra overhead caused by cooperator RTC responses and destination NACK transmissions, which are more noticeable in lower traffic loads. As λ grows, energy efficiency in each scheme converges to its own steady state.

**Figure 11:**
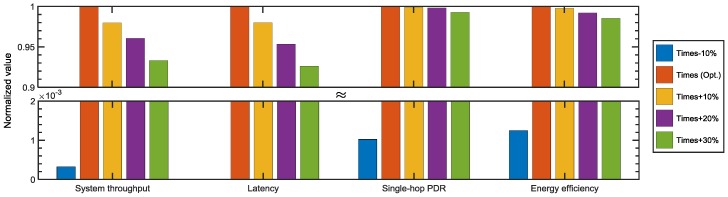
Performance variation with different lengths of the waiting times ($N_{\mathrm{coop}}^{\max}=2$, DATA size = 1 kbits, λ=0.01 ).

Figure 11 verifies that the waiting times provided in the manuscript guarantee the highest network performances in UCMAC. In the figure, each result of the performance metrics is normalized to result of the waiting times (orange-colored bar) obtained through the Equations (2)–(10) that could potentially yield the best network performances. Adding extra time of 10–30% to the calculated waiting times degrades the performances of the network only by a small margin. That is largely due to the waste of the channel resources. On the other hand, with a 10% reduction of the waiting times (blue-colored bar), the network’s performance is significantly degraded. That means the values we got from the equations provide sufficient, but not excessive time for the desired packets to arrive. As a result, the maximum cooperative diversity gains require a careful decision of the waiting times, which compensates for the effects of the space–time uncertainty problem.

## 6. Conclusions

This paper proposes a cooperative MAC protocol for UWSNs named UCMAC. For the purpose of improving network capabilities in the error-prone underwater channel, UCMAC builds spatial diversity using cooperative retransmission. In addition, to minimize extra overheads caused by this cooperation, the neighbors located at more advantageous positions for retransmission than the source selectively participate in the cooperation. Also, the order of preferred cooperators, based on closeness to the destination, is appended to data without extra packet exchange. Moreover, this scheme avoids the packet collisions that frequently occur in previous cooperative communication schemes by designating the most preferable cooperators one by one. Simulation results showed that UCMAC outperforms comparable schemes, including MACA-U and CD-MACA, in terms of system throughput, latency, single-hop PDR, and energy efficiency, alleviating the space–time uncertainty problem. We are currently planning additional research to include the cross-layer approach of establishing a new cooperator selection criterion that adapts to underwater channel quality.

## Figures and Tables

**Table 1:** Notations used to explain UCMAC.

Symbol	Description
$T_{x}$	Transmission time of a packet x ^1^
$\tau_{i,j}$	Propagation delay between nodes *i* and *j*
$\tau_{\max}$	Maximum propagation delay
$\tau_{\mathrm{back}}^{C_{j}}$	Backoff time of a cooperator *j*
$N_{\mathrm{coop}}^{\max}$	Maximum number of cooperators allowed in one session
$d_{\mathrm{delay}}^{S}$	Delay of DATA transmission at a source
$d_{w\_x}^{i}$	Duration that a node *i* waits for reception of a packet x ^1^
${\mathbb{N}}_{\mathrm{coop}}$	List of cooperators recognized by a source
${\mathbb{N}}_{\mathrm{neg}}$	List of potential RTC–CTS collision-causing neighbors

^1^*x* ∈ {RTS, RTC, CTS, DATA, NACK, ACK}.

**Table 2:** System parameters for simulation.

Parameter	Value
Grid size	1 km×1 km
Propagation speed	1500 m/s
Transmission range	2500 m
Data rate	2400 bps
Tx Power	20 W
Rx power	756 mW
Maximum number of RTS transmission	5
Control packet size ^1^	120 bits

^1^ RTS, RTC, CTS, NACK, ACK packets.
